# Plant-growth-promoting bacteria from rhizosphere of Chilean common bean ecotype (*Phaseolus vulgaris* L.) supporting seed germination and growth against salinity stress

**DOI:** 10.3389/fpls.2022.1052263

**Published:** 2022-12-22

**Authors:** Cynthia Meza, Francisca Valenzuela, Alex Echeverría-Vega, Aleydis Gomez, Shrabana Sarkar, Ricardo A. Cabeza, Ariel D. Arencibia, Karla Quiroz, Basilio Carrasco, Aparna Banerjee

**Affiliations:** ^1^ Doctorado en Biotecnología Traslacional (DBT), Facultad de Ciencias Agrarias y Forestales, Universidad Católica del Maule, Talca, Chile; ^2^ Centro de Biotecnología de los Recursos Naturales (CENBio), Facultad de Ciencias Agrarias y Forestales, Universidad Católica del Maule, Talca, Chile; ^3^ Centro de Estudios en Alimentos Procesados (CEAP), Talca, Chile; ^4^ Centro de Investigación de Estudios Avanzados del Maule, Vicerrectoría de Investigación y Posgrado, Universidad Católica del Maule, Talca, Chile; ^5^ Plant Nutrition Laboratory, Department of Crop Sciences, Faculty of Agricultural Sciences, University of Talca, Talca, Chile

**Keywords:** abiotic stress, *Bacillus*, plant growth, plant growth promoting bacteria (PGPB), salinity

## Abstract

Salinity abiotic stress is increasing day by day due to continuous global warming and climate change. This is also becoming one of the major causes behind the reduction in crop production. Plant–bacteria interaction plays an essential role in improving crop yield without using any chemical fertilizers. The present study aims to characterize the interaction between plant-growth-promoting bacteria (PGPB) and their role in mitigating salinity stress for local variety crops. Therefore, in this work, two PGPB, namely, *Bacillus proteolyticus* Cyn1 and *Bacillus safensis* Cyn2, were isolated from rhizospheric soil of the Chilean common bean ecotype “Sapito” (*Phaseolus vulgaris* L.), and their PGPB traits were analyzed. Cyn1 can produce NH_3_ and HCN and also secrete siderophores, whereas Cyn2 produced NH_3_ and siderophores but responded negatively to HCN production. Both the isolated bacteria have shown a positive result for ACC deaminase production, phosphate solubilization, and catalase enzyme secretion. Under all three tested abiotic stresses, i.e., temperature, water, and salinity, both the bacteria and their consortium have demonstrated positive responses. Cyn1 under temperature stress and water stress can produce a biofilm network to combat the stress. While under salinity stress, both the PGPB isolates indicated the production of stress components and cytoplasmic inclusion bodies. Based on the response, among all other abiotic stresses, salinity stress was chosen for further plant–bacteria interaction study and growth. Visible root colonization of the bacteria has been observed in comparison to the control. The germination index was 100% for all experimental setups of seed bacterization, both under control conditions and salinity stress. Both bacteria responded with good PGP traits that helped in the growth of healthy plants after the bacterial treatment in final pot experiments. Additionally, the consortium and the plants treated with Cyn1 have demonstrated high production of photosynthetic pigments in both experimental setups. Both *B*. *proteolyticus* Cyn1 and *B*. *safensis* Cyn2 have shown promising PGP characters and efficient response against toxicity related to salinity. Hence, both of these bacteria and consortium can be used for improved agricultural production of Chilean native common beans in the near future.

## Introduction

Common bean (*Phaseolus vulgaris* L.) is considered to be one of the most consumed leguminous crops worldwide (Castro-Guerrero et al., 2016). In Latin America, it is the main staple food in people’s diet because of its high nutritional qualities (Torres et al., 2017), with a range of protein ~16%–33% depending on different landraces (Luna-Vital et al., 2015). As a legume, being associated with nitrogen-fixing bacteria, the common bean is also considered to be eco-friendly. It reduces the use of synthetic fertilizers, which is the key for a sustainable agriculture (Castro-Guerrero et al., 2016). Common beans are indigenous to Mexico/Central America and South America, leading to a historical domestication nearly ~8,000 years ago. This has driven to an adaptation towards Mediterranean environments in two important gene pools, Mesoamerican and Andean, respectively (Castro-Guerrero et al., 2016). Local common bean landraces found in Chile belong to the Andean pool (Blair et al., 2007). Chilean ecotypes of *P. vulgaris* L. are considered to be a subcenter of genetic diversity, as its ecotypes have characteristics that are not found in the germplasm of other Andean landraces, *viz*., Nueva Granada and Peru. Among ~200 different common bean landraces of Chile known to date, the most representative types are Tortola, Coscorron, Manteca, Bayo, Araucano, Peumo, and Sapito (Bascur and Tay, 2005). However, the biodiversity of local Chilean bean varieties has eroded in the last three decades due to the combination of several factors such as the decrease in small family farming, increase in production costs, rise in imports of vegetable proteins, and the use of arable land for the production of fruit crops, among others (Baginsky and Ramos, 2018). On the other hand, due to the biotic and abiotic stresses to which they have been subjected over time, ancestral varieties have been replaced by resistant modern varieties.

During the ongoing global warming and climate change events, agricultural soil in areas with a Mediterranean climate is continuously affected by increased temperature, drought, and soil salinization (Jacobsen et al., 2012). These are the main types of abiotic stresses causing adverse effects on crop growth and productivity worldwide. As per example, high temperature stress reduces yield such as the number of spikes and florets for rice plant and sorghum. Even a high temperature leads to drought followed by salinity stress in the soil (Dawood et al., 2020). One research also found that salinity and drought occur simultaneously to induce more damage, including germination to crops rather than a single stress (Dawood et al., 2021). A decrease in temperature along with an increase in salinity may cause delayed germination, too (Kayani and Gul, 2000). The excessive presence of salts in the soil is a problematic factor responsible for the reduction of plant growth and crop productivity (Jamil et al., 2011). For example, in the year 2018, due to a change in the water flow of the Mataquito River, the Chilean Maule region got contaminated with saline water from the Pacific Ocean, which resulted in the farmers losing bean crops irrigated with saline water in the surrounding valley. It is estimated that more than 1 billion hectares of land are affected around the world by soil salinization. In addition, climate change will increase the area of semi-arid and saline lands due to a reduction in rainfall (Martínez-Villavicencio et al., 2011). The effects of abiotic stress (drought, temperature, and salinity) in *P*. *vulgaris* L. induce oxidative pressure that has been reported earlier *via* the presence of stress indicators such as H_2_O_2_, TBARS, glutathione, ascorbic acid, and proline. It has also been found that drought-tolerant wheat plants try to accumulate proline, soluble sugars, and photosynthetic pigments (chlorophyll a and b) resulting in an increased antioxidant activity (Sallam et al., 2019). In addition, an increase in antioxidant enzymes peroxidase, β-amylase, and acid phosphatase is reported. All these affect the growth rate and fresh mass (Babu and Devaraj, 2008). Drought stress is also reported to have a severe effect on common bean farming *via* reduced total biomass and seed yield, impaired photosynthate translocation, partitioning, decreased number of pods and seeds per plant, declined root length and mass, and increased overall maturation time (Darkwa et al., 2016). Apart from these, *P*. *vulgaris* L. is reported long back to tolerate salinity stress to a level but not high salinity. Actually, among the annual crops, beans are among the most sensitive to salinity (1 m mhos/cm). At low salinity stress, initial developmental delay or morphogenetic shift of the leaves may be observed. Salinity affects shoot growth more than root growth. Moreover, farmers choose to use chemical fertilizers for quick crop yield, causing an alteration in the balance of the native soil microbiota. This, in turn, results in the bioaccumulation of toxic compounds in soil, decreased soil organic matter, soil hardening, groundwater contamination, and pH alteration, and, in extreme cases, makes the land derelict (Pastor-Bueis et al., 2019). Studies related to phytohormones for salt stress elimination have been performed before. For example, jasmonic acid (JA) (60 µM) priming not only reinforces defense strategies against salinity stress (14.50 dSm^−1^ NaCl) in wheat cultivars (ZM9 and YM2) but also causes increase in vegetative growth and crop yield (Sheteiwy et al., 2022). The use of rhizosperic bacteria or consortium to combat salinity stress in Chilean common bean had not been studied earlier.

Plant–microbe interaction plays a fundamental role in improving crop yield and establishing a new strategy to reduce the use of chemical fertilizers. The basis of this symbiotic interaction is the soil microorganisms (plant-growth-promoting bacteria or PGPB) that colonize or live around the soil rhizosphere. They actively promote nutrient uptake, encapsulate salts, mineralize organic matter, and promote plant growth, *via* increased absorption and availability of nutrients from the soil. This develops defense mechanisms against various biotic stress of pests and pathogens and also helps to withstand high concentrations of salt, temperature, and water stress (Moreno Reséndez et al., 2018). These microorganisms present in the rhizospheric region may react with different metabolites either positively, negatively, or neutrally in order to influence nutrient uptake and plant growth. In case of positive interaction, growth regulators mimic cross-signaling between different species of rhizospheric bacteria. This mechanism of interaction helps in nutrient uptake (Yadav et al., 2015). The most important component of commercial biofertilizer is PGPB, which is eco-friendly, cheap, more accessible, more efficient, and productive in nature (Kumari et al., 2018). These reasons make it necessary to isolate PGPB from common beans root. Moreover, the plant–microbe interaction of Andean gene pool of common beans, more specifically Chilean ecotypes, has not been studied earlier. Among many PGPB strains (*Pseudomonas*, *Azobactor*, *Azospirillum*, etc.), one of the largest bacterial genera, *Bacillus*, has elevated capacity to induce abiotic stress tolerance in crop plant. In addition, under all kinds of abiotic stresses (temperature, drought, and salinity), *Bacillus* spp. have been found to improve plant growth, alleviate the toxicity associated with abiotic stresses, improve phosphate uptake, and better chlorophyll content (Ali et al., 2022; Abdelmoteleb and Gonzalez-Mendoza, 2020). Therefore, in this work, our objective was to characterize PGPB from the rhizospheric soil of Chilean *P. vulgaris* L. ecotype and to study their role in seed germination and promoting plant growth under salinity stress conditions.

## Materials and methods

### Collection of rhizospheric soil sample

**Figure 1:**
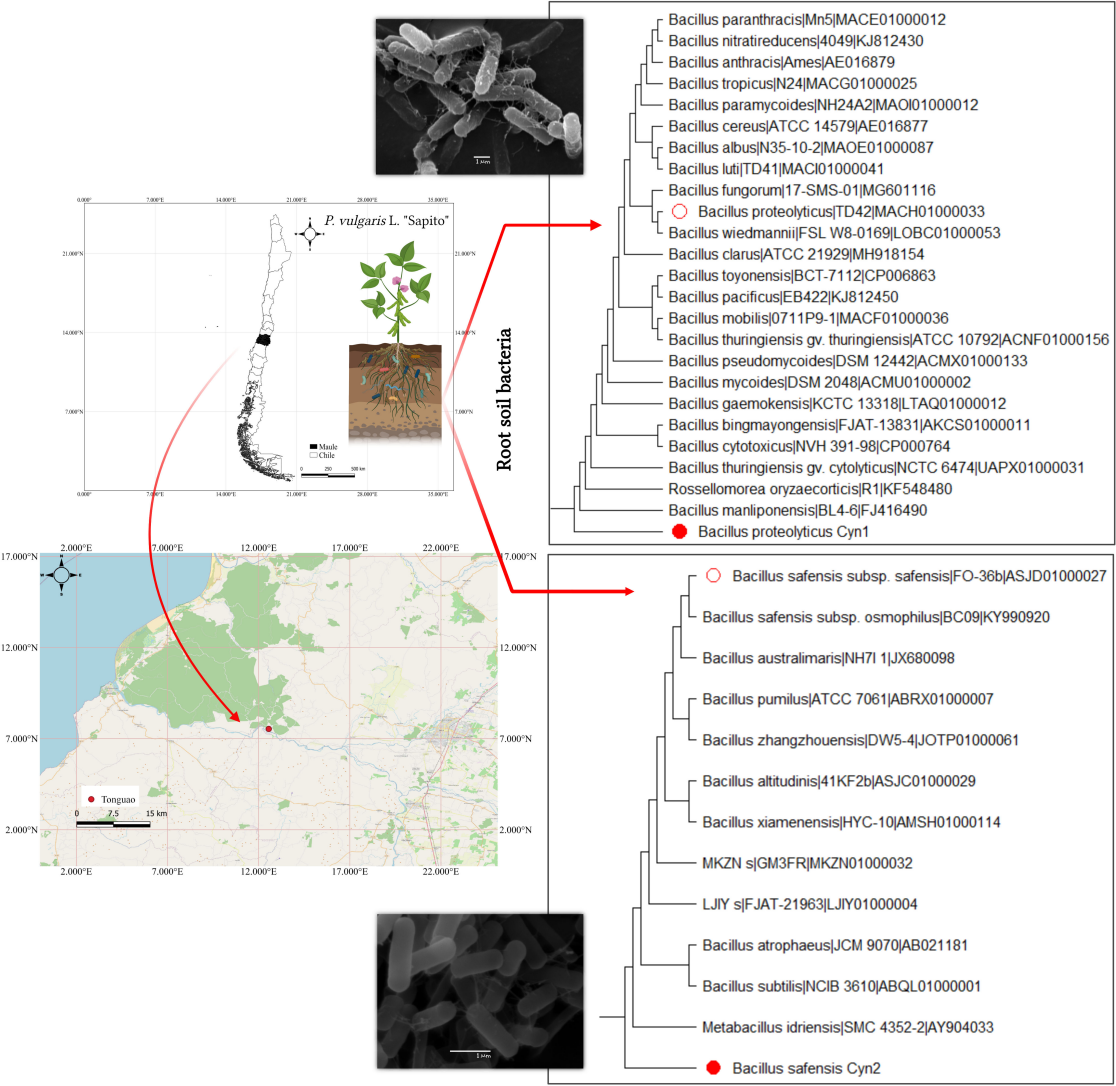
Study maps showing the location of the rhizosphere soil (*P*. *vulgaris* L. “Sapito”) collected from the Tonguao area of Maule region, Chile. On the right, the maximum likelihood phylogeny with 1,000 bootstrap value of the isolates Cyn1 and Cyn2 (filled red circles) showing similarity with *B. proteolyticus* and *B. safensis* respectively, (outlined red circle) with scanning electron micrograph images of the bacterial isolates in respective insets.

The rhizospheric soil samples from the Chilean ecotype of *P*. *vulgaris* L. (Sapito variety common bean, local name “poroto Sapito”) was collected in April 2021 from the agricultural land of a local family located in Tonguao (latitude: −35.4236°S; longitude: 72.0563°W), Maule region of Chile (**Figure 1**). The plants were uprooted along with the soils and brought to the laboratory in sterile, zip-locked bags for further studies. The non-rhizospheric soil and the large soil aggregates were removed. Only the soil that adhered to the roots was separately collected from the plant to get a rhizospheric soil sample. Soil sample was kept moist in the dark at 4°C for further isolation of bacteria. From the collected soil sample, 1 g of rhizospheric soil was used for further experiments.

### Isolation and identification of plant-growth-promoting bacteria (PGPB)

The PGPB were isolated from the collected soil samples *via* serial dilution method in Luria–Bertani (LB) agar medium (BD DIFCO, New Jersey, USA) supplemented with 1.8% agar. A 100 μl of appropriate dilution of the soil sample was plated on LB agar and was incubated for 24 h at 28°C. The distinct colonies grown on the plates were purified by subculturing. The isolates were maintained on LB medium for further experiments.

In order to genetically identify the PGPBs, amplification of the isolated DNA was carried out using universal 27F and 1492R primers for the 16S rRNA gene. The amplicons were sequenced by capillary electrophoresis in Macrogen Inc. (Seoul, South Korea). Once the sequences were obtained in both directions, they were assembled and refined with Secuencher 5.4.6 software (Gene Codes Cooperation, MI, United States) to obtain the consensus sequences. Then EzBioCloud database (accessed on 12-01-2022) was used to carry out a local alignment. Evolutionary distances between the sequences were calculated (Tamura and Nei, 1993), and the phylogenetic tree was prepared following the maximum likelihood method with a 1,000 bootstrap value using MEGA 11 (Tamura et al., 2021) followed by deposition in GenBank (https://www.ncbi.nlm.nih.gov/; accessed on 26-8-2022).

### Characterization of PGPB traits

#### Production of ammonia, hydrogen cyanide, and siderophores

Traditional methodology for the characterization of PGPB traits (production of ammonia, hydrogen cyanide, and siderophores) have been followed as described before by Gupta and Pandey (2019) with some little modifications. First, bacterial isolates were grown in peptone water broth (peptone: 10 g L^−1^, NaCl: 5 g L^−1^) using an incubator with orbital shaker (YIHDER LM-510D, Xinbei, Taiwan) at 150 rpm for 24 h at 28°C. Then, in order to check the production of ammonia (NH_3_), 100 μl of Nessler’s reagent was added to 3 ml of bacterial culture (in the early stationary phase) to notice the change of the color of the media. A slight change in media color from yellow to brown color is considered positive for NH_3_ production (Gupta & Pandey, 2019). To verify the production of hydrogen cyanide (HCN), the bacterial isolates were grown in King’s B medium (TM Media, Rajasthan, India) supplemented with 0.4% (w/v) glycine with a Whatman filter paper saturated with alkaline citrate buffer placed on the upper lids of the Petri plates. The plates were further incubated for 4 days at 28°C for a reddish-brown appearance as the indicator of positive HCN production (Gupta & Pandey, 2019). For siderophore production, the PGPB isolates were inoculated on Chrome Azurol S (CAS) agar plates and incubated for 4 days at 28°C. An orange-yellow halo around the bacterial colony indicated positive siderophore production.

#### 1-Aminocyclopropane-1-carboxylic acid deaminase and catalase activity

For ACC deaminase activity determination, both the bacterial isolates were cultured in Dworkin and Foster (DF) minimal salts medium containing KH_2_PO_4_ 4 g L^−1^, Na_2_HPO_4_ 6 g L^−1^, MgSO_4_ • 7H_2_O g L^−1^, glucose 2 g L^−1^, gluconic acid 2 g L^−1^, and citric acid with trace elements FeSO_4_ • 7H_2_O 1 mg, H_3_BO_3_ 10 mg, MnSO_4_ • H_2_O 11.19 mg, ZnSO_4_ • 7H_2_O 124.6 mg, CuSO_4_ • 5H_2_O 78.22 mg, MoO_3_ 10 mg, and agar 15 g L^−1^, pH 7.2, with additional 3 mM ACC as the only source of nitrogen at 30°C for 7 days in an incubator (Biobase BJPX - H50, Jinan, China). The colonies that grew on the plates were considered as producers of ACC deaminase enzymes as stated before by Gupta and Pandey (2019). For the detection of catalase-enzyme-producing activity, a loop full of the bacterial isolates was placed on separate slides with a drop of hydrogen peroxide (H_2_O_2_) added on it. The appearance of bubbles was considered to be a positive production of catalase enzymes.

#### Phosphate solubilization

The experimental method was done as described before by Gupta and Pandey (2019) with little modification. Both the bacterial isolates were cultured in a modified Pikovskaya agar medium (TM Media, Rajasthan, India) supplemented with 2% (w/v) tricalcium phosphate (TCP) and incubated at 30°C for 7 days in an incubator (LM-450D BIOBASE, Jinan, China). The development of clear zones around the bacterial colonies were considered as positive phosphate solubilization reaction by the specific bacterial isolates.

#### Biofilm formation

Observing the ropy/mucoid colony appearance, a characteristic feature of biofilm production, a biofilm formation study was performed for both the bacterial isolates as earlier reported by Marín-Sanhueza et al. (2022) with some small modifications. For this, cells were inoculated in 50 ml LB medium (Difco™, NJ, USA) for 24 h at 30°C with continuous shaking of 150 rpm (LM-450D BIOBASE, Jinan, China). A 50 μl of logarithmic phase culture (10^8^ CFU ml^−1^) was transferred to 96-well microtiter plates along with 250 µl of fresh culture broth (1:5 v/v). The microplate was further incubated for 24 h at 30°C (BJP-H50 BIOBASE, Jinan, China). Every 24 h, the planktonic cells were removed from the microplate and fresh broth was added (1:5 v/v). In this way, the biofilm was maintained for 4 days, as visible appearance of the biofilm came to the microplate. The viability of the surface-associated bacterial cells in the biofilm matrix was observed *via* staining with LIVE/DEAD™ BacLight™ (Thermo Fisher Scientific, MA, USA). SYTO 9 green only stains live cells in a fresh sample, whereas propidium iodide red only stains dead cells and extracellular biofilm DNA (eDNA). The stained cells were observed with a Leica Stellaris 5 confocal microscope (Leica Microsystems, Wetzlar, Germany) with excitation/emission for SYTO 9 of 485 nm/498 nm and propidium iodide of 535 nm/617 nm.

### Tolerance to abiotic stress

Both the different bacterial isolates and their consortium (logarithmic phase, 10^8^ CFU ml^−1^ culture in 1:1 v/v proportion) were observed for tolerance to distinct abiotic stresses, *viz*., increasing temperature, induced drought, and salinity.

#### Temperature stress

Both the bacterial samples were cultured in 50 ml of LB medium at 30°C and 150 rpm for 24 h. The cultures were subjected to brief and increasing temperature treatment of 40, 50, 60, 70, 80, 90, and 100°C for 15 min. Then, the temperature-treated bacterial cultures were subcultured for 24 h at 30°C. Visible bacterial growth was determined to be temperature tolerant.

#### Water stress

To determine the tolerance of the bacteria under induced drought conditions, 20 ml of liquid LB medium was supplemented with increasing concentrations of polyethylene glycol (PEG 8000): 5%, 10%, 15%, 20%, and 25%. The bacteria were cultured at 150 rpm at 30°C for 24 h. Bacterial growth was measured in a Mobi absorption spectrophotometer at a wavelength of 590 nm (μ2 MicroDigital, Seoul, South Korea).

#### Salinity stress

Salt tolerance of the isolated bacteria were confirmed by observing the growth in salt-supplemented media. For this, bacteria were inoculated in LB agar medium supplemented with 1%–10% NaCl (with a 1% interval between each salt concentration) for 72 h at 30°C. Visible bacterial growth indicated its tolerance to different salinity levels.

Cellular morphology of the two different bacterial isolates at each stress treatment was analyzed using scanning electron microscopy (SEM). For this, 1 ml of bacterial culture was centrifuged at 10,000 rpm, 4°C for 5 min, and the supernatant was discarded. The pellet was washed several times with 0.1 M phosphate buffer (pH 7.2) and fixed with 2.5% glutaraldehyde solution. The fixed samples were placed on a 10-mm carbon grid and further have been gold-coated (SPI Supplies, West Chester, PA, USA) to make it conductive. Then, the sample was observed under a scanning electron microscope (JEOL JSM 6380LV, Tokyo, Japan). Furthermore, to determine the cellular viability of each treatment under different abiotic stresses, confocal microscopy has been used. For this, 3 μl of propidium iodide and SYTO 9 stains from LIVE/DEAD (BacLight^TM^) kit were diluted (1:10 v/v) for each sample and observed with a Leica Stellaris 5 confocal microscope (Leica Microsystems, Wetzlar, Germany) with excitation/emission for SYTO 9 of 485 nm/498 nm and propidium iodide of 535 nm/617 nm. Additionally, intracellular changes under salinity stress have been observed *via* transmission electron microscopy (TEM). For this, fresh bacterial cells under salinity stress were fixed on carbon-coated grid by negative staining with 2% uranyl acetate and observed under a TEM (Libra 120 Plus, Carl Zeiss, Oberkochen, Germany).

Based on the results obtained from all the three different abiotic stress treatments (i.e., temperature, water, and salt stress screening), salinity stress was further chosen for seed bacterization, germination, and pot experiments.

### Seed bacterization and germination

Seeds of the local “Sapito” variety of *P*. *vulgaris* L. were collected from Centro de Estudios en Alimentos Procesados (CEAP), Talca, Chile. The healthy and uniform seeds were washed with distilled water thoroughly. Then, surface sterilization of those seeds was done using a 2% sodium hypochlorite treatment, followed by rinsing with distilled water. The bacterized seeds were germinated in two experimental sets with two different treatments: set 1, seeds germinated in distilled water and set 2, seeds germinated in 5% aqueous NaCl solution as salinity stress. The percentage of NaCl has been chosen based on the lower tolerable concentrations of both the bacterial isolates for their equal functioning in the consortium. In each set, three experimental conditions were applied for seed bacterization: condition 1, experimental sets treated with Cyn1 bacteria; condition 2, experimental sets treated with Cyn2 bacteria; and condition 3, experimental sets treated with a consortium of Cyn1 and Cyn2 as mentioned before. This entire experiment was performed in triplicates. For experimental set 1, the solution for seed bacterization consisted of distilled water along with bacteria (logarithmic phase, 10^8^ CFU ml^−1^) at a ratio of 10:1. Whereas for experimental set 2, the same bacterial concentration was mixed with 5% aqueous NaCl solution in a similar ratio for inducing salinity stress in the bacterized seeds. The seeds were allowed to germinate in the dark at 24°C for 3 days. After that, both the sets with germinated seedlings were placed in normal photoperiod of 16 h light and 8 h darkness for 2 days in a plant growth chamber at 27°C for further growth. After 2 days, before transfer in the pot experiment, plant growth parameters such as plumule length, radicle length, root length, number of roots, shoot length, number of leaves, width of leaves, germination rate, relative germination, % toxicity, tolerance index, and vigor index were recorded as mentioned before by Sarkar et al. (2021). The values were added to an Excel of the Microsoft^®^ 365 office package, and standard deviations were made. For the assessment of seed germination-related parameters, the following equations (**Equations 1–4**) were considered:


(1) $$Relative\;germination=\frac{\text{number of gemination in each treatments}}{\text{number of gemination in control}}\times 100$$

(2) $$\%\;of\;toxicity=\left[\frac{\left\{\left(radicle\;length\;of\;control-radicle\;length\;of\;treatment\right)\right\}}{radicle\;length\;of\;control}\right]\times 100$$

(3) $$Tolerance\;index=\frac{Mean\;length\;of\;longest\;root\;in\;tratment}{Mean\;length\;of\;longest\;root\;in\;control}$$

(4) Vigor index={(mean root length+mean shoot length)}×%of germination

Furthermore, the plant–bacteria association 5 days post-bacterization has been observed *via* confocal microscopy. The cellular viability post-salinity stress and root colonization have been visualized, too. For this, LIVE/DEAD BacLight™ kit was used as mentioned before and observed with a Leica Stellaris 5 confocal microscope (Leica Microsystems, Wetzlar, Germany) with excitation/emission for SYTO 9 of 485 nm/498 nm and propidium iodide of 535 nm/617 nm.

Permutational analysis of variance (PERMANOVA) was performed to evaluate the significance of factors medium (with two levels: H_2_O and NaCl) and test (with four levels: Cyn1, Cyn2, consortium, and control) nested in the medium.


(5) Y= x + Me + Te (Me) + e

Variables were normalized, and a resemblance matrix was constructed based on the Euclidian distance of the samples. Draftsman plot is a scatter plot showing the interrelation between variables in multivariate data. A hierarchical cluster analysis was constructed based on Pearson’s coefficients to visually identify groups of variables and as a tool to help choose a representative variable for each high-correlated cluster. Pearson’s coefficient was calculated to determine the correlation between pairs of variables. A high correlation (>0.85) was set as the maximum. If the correlation value was higher (or lower in case of negative), one of the variables was left out for the next statistics and PCA plots. Principal component analysis was done to reduce the dimensionality of datasets, increasing interpretability and avoiding information loss as much as possible. A normalized table was used as the source of data. Selected variables for this analysis (avoiding highly correlated variables) were total length, plumule length, radicle length, number of roots, node length, and leaves length. All the analyses were made with the help of Primer6 + PERMANOVA add-on (Primer-e) software as described before by Anderson et al. (2008).

### Pot experiments

A pot experiment was carried out after germination of bacterized seeds. For this, each germinated seedlings were placed in different pots (2 L volume, 13 cm × 13 cm × 13 cm) containing 50% sand and 50% humus (1:1 v/v, 1.5 kg) and placed in a plant growth chamber at a constant temperature of 24°C and 50% relative humidity. Each experimental pot was kept under a well-watered condition (each 2 days, 50 ml) in photoperiods of 16 h light (light intensity of 340 μmol m^−2^ s^−1^) and 8 h darkness for 1 month until the onset of flowering. Pots of set 1 were treated with normal water, whereas pots of set 2 were irrigated with 5% aqueous NaCl solution to maintain the salinity stress. The entire pot experiment was performed in triplicates.

After a month of growth, photosynthetic pigments of the differently treated plants were measured. For this analysis, the total leaves of each plant were collected from each set of experiments, and 0.1 g of the total leaves was added to a mortar with 5 ml of methanol. It was macerated and filtered with a 0.45-µl polyvinylidene fluoride (PDVF) filter. Furthermore, it was centrifuged (BOECO C-28A, Hamburg, Germany) at 4,000 rpm for 1 min. The supernatant was transferred to a microplate, and the absorbance was measured using a MOBI microplate spectrophotometer (μ2 MicroDigital, MOBI, Seoul, South Korea) at a wavelength of 662, 646, and 470 nm, respectively, for chlorophyll a (C_a_), b (C_b_), and carotenoids as stated before by Dere et al. (1998). The entire experiment has been performed in triplicate. With the values, the means and the standard deviations were made in an Excel using the Microsoft^®^ 365 office package. The concentration of chlorophyll a, b, and total carotenoids were calculated with the formula mentioned in **Equations 6–8** accordingly and have been presented in an interactive heatmap *via* Heatmapper server (Babicki et al., 2016). The equations are given below:


(6) $$Ca=15.65\times A_{662\;}-7.340\times A_{646}\;$$

(7) $$Cb=27.05\times A_{646\;}-11.21\times A_{662}\;$$

(8) $$Total\;carotenoids=\frac{1,000\times A_{470\;}-2.860\times Ca-129.2\times Cb}{245}$$

## Results

### Isolation and identification of plant-growth-promoting bacteria

From collected rhizospheric soil samples of *P*. *vulgaris* L. (“Sapito” variety common bean), two bacteria have been isolated, namely, Cyn1 and Cyn2. According to the 16S rRNA identification using the EZ-Taxon server, it has been found that the isolate Cyn1 demonstrated 100% nucleotide identity with the type strain *Bacillus proteolyticus* TD42, whereas Cyn2 has 99.86% nucleotide identity with *Bacillus safensis* subsp. *safensis*. Therefore, Cyn1 was identified as *B*. *proteolyticus* Cyn1, and isolate Cyn2 was identified as *B*. *safensis* Cyn2 (**Figure 1**). The sequences were submitted in GenBank with the accession numbers OM247622 and OM247623 for Cyn1 and Cyn2, respectively. The isolates were further maintained on LB medium for other experiments.

### Characterization of PGPB traits

#### Production of NH_3_, HCN, and siderophores

The entire PGPB traits and their characteristics demonstrated by both the isolates have been described in **Table 1**. Both the isolated bacteria showed positive production of NH_3_ by colorimetric detection. *B. proteolyticus* Cyn1 showed a darker brownish appearance compared to *B. safensis* Cyn2, which developed a faint brown color; hence, *B. proteolyticus* Cyn1 was determined as the maximum producer of NH_3_ among the two isolates. *B. proteolyticus* Cyn1 produced HCN, confirmed by the change in color of the filter paper from yellow to reddish brown. The color change was not found in the HCN detection experiment performed for *B. safensis* Cyn2, which indicated that Cyn2 is a negative HCN producer, but Cyn1 is positive for the test. For siderophore production experiment, a yellow-colored halo around the bacterial colonies grown in CAS agar plates has been observed for both the isolated bacteria, *B. proteolyticus* Cyn1 and *B. safensis* Cyn2. This result indicates that both Cyn1 and Cyn2 can produce siderophores (**Table 1**).

**Table 1:** Plant-growth-promoting (PGP) traits and abiotic stress tolerance of the bacteria isolated from root rhizosphere of *P*. *vulgaris* L. “Sapito.”.

PGP characteristic	*B*. *proteolyticus* Cyn1	*B*. *safensis* Cyn2
**Ammonia (NH_3_) production**	+++	++
**HCN production**	+++	−
**Siderophore production**	++	+
**ACC deaminase production**	+	+
**Catalase activity**	++	+++
**Phosphate solubilisation**	+++	+++
**Biofilm formation**	++	+++
**Temperature tolerance (°C)**	80	70
**Drought tolerance (% PEG)**	10	10
**% Salt tolerance**	5	8

(+++) highly positive result, (++) moderately positive result, (+) weakly positive result, and (−) negative result.

#### ACC deaminase and catalase activity

Two bacterial isolates, Cyn1 and Cyn2, were screened for ACC deaminase activity based on the enrichment method in DM minimal salt medium, where ACC was used as the sole nitrogen source. Both the tested organism grew well on minimal salt medium supplemented with ACC serving as the sole nitrogen source. Therefore, the result depicts that both bacteria are positive ACC deaminase producers (**Table 1**). Both the isolated bacteria *B. proteolyticus* Cyn1 and *B. safensis* Cyn2 also tested catalase positive. While a vivid appearance of oxygen bubbles has been observed for *B. safensis* Cyn2, the bubble was comparatively less for *B. proteolyticus* Cyn1 (**Table 1**).

#### Phosphate solubilization analysis

Both the PGPB isolates, Cyn1 and Cyn2, were screened for phosphate solubilization activity using Pikovskaya agar medium (TM Media, Rajasthan, India) where TCP was used as the sole carbon source. A clear halo of phosphate solubilization around the colonies of both the bacterial isolates Cyn1 and Cyn2 was observed, which indicates a positive result. As it may be observed from **Table 1**, both the PGPB isolates *B. proteolyticus* Cyn1 and *B. safensis* Cyn2 are high solubilizers of phosphates.

#### Biofilm formation

**Figure 2:**
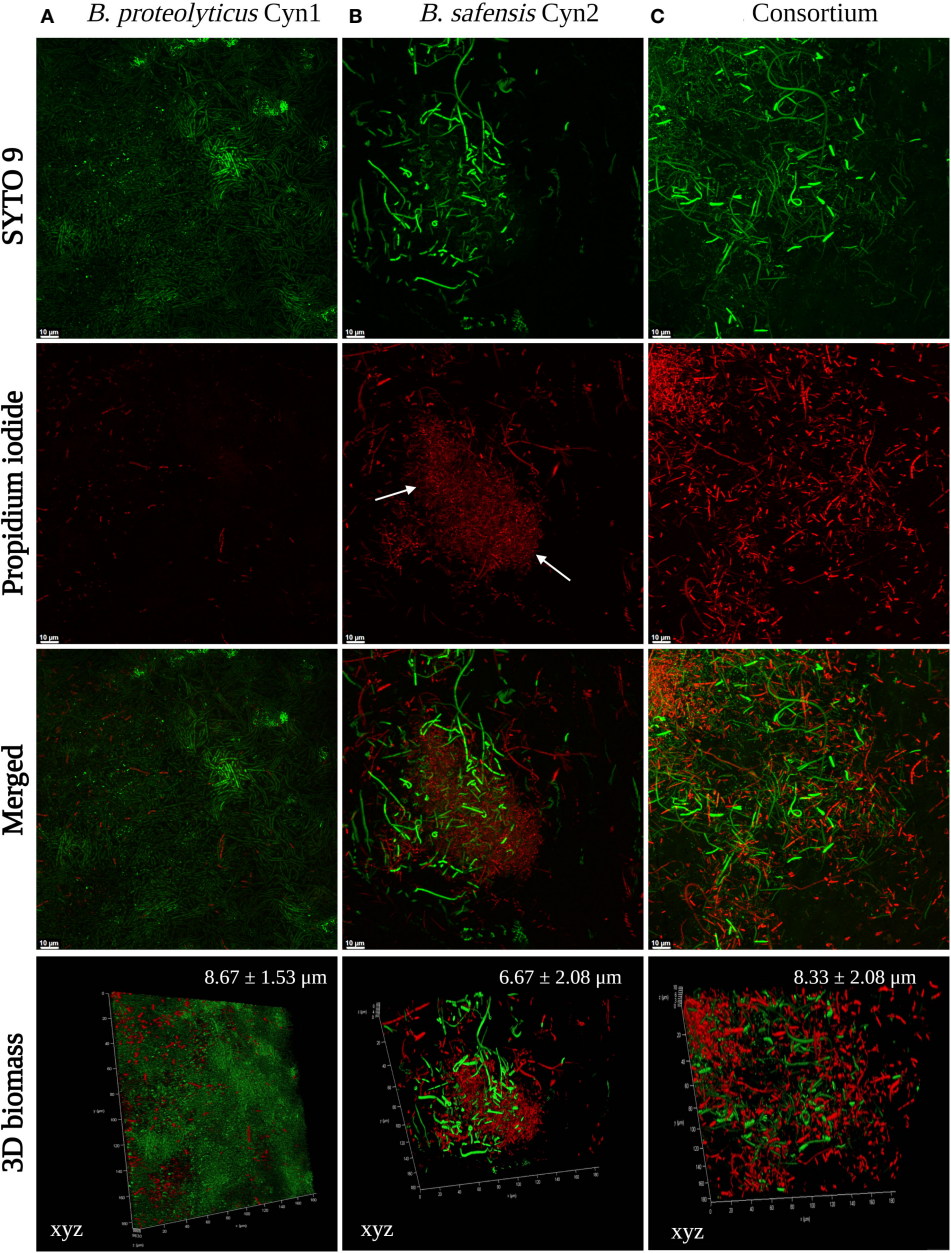
Confocal microscopy images of **(A)** *B. proteolyticus* Cyn1, **(B)** *B. safensis* Cyn2, and **(C)** consortium biofilms. Staining was done with SYTO 9/propidium iodide (LIVE/DEAD BacLight Bacterial Viability kit), and images were acquired at 60 x magnification. Only live cells internalize SYTO 9 (fluorescing green), whereas dead cells allow the uptake of PI (fluorescing red). In all panels, the results are representative images of three independent experiments and biofilm growth was evaluated at 96 h. Three-dimensional (3D) biomass of the biofilm for each set is also represented in the lower panels. The white arrows indicate the presence of eDNA.

The formation of a vivid biofilm network has been observed in the case of *B. safensis* Cyn2 and the consortium, as may be observed in **Figure 2**. Additionally, a mucoid colony phenotype has also been observed for both PGPB isolates in LB agar medium, which indicated biofilm formation capacity, too (**Supplementary Figure S1**). Most of the cells of *B. proteolyticus* Cyn1, *B. safensis* Cyn2, and their consortium have been observed to consist unaltered membrane integrity, emitting green fluorescence of SYTO 9. While *B*. *proteolyticus* Cyn1 majorly emit green fluorescence with the highest bacterial count among the three, *B*. *safensis* Cyn2 is observed with lower bacterial count compared to Cyn1 but with long chains of non-motile bacilli cells, a characteristic feature of bacilli biofilm. The red fluorescence of isolate Cyn2 emerges from both dead or damaged bacterial cells and eDNA in the biofilm matrix. However, bacterial chaining in the *in vitro* biofilm network may also be observed to some extent in Cyn1, which indicated structured communities in the form of microcolonies in the case of both Cyn1 and Cyn2. The consortium clearly demonstrated increased bacterial count and the presence of both Cyn1 and Cyn2 isolates in the network. The presence of long non-motile bacterial chains, some damaged or dead bacteria, mostly viable bacterial cells, and the presence of eDNA in the biofilm matrix have been observed in all the cases. The consortium biofilm indicates synergistic biofilm network development by both the isolates Cyn1 and Cyn2 with clearly visible small Cyn1 chains and long green fluorescing Cyn2 chains with eDNA in the biofilm matrix. The highest 3D cellular biomass formed by Cyn1 was of 8.67 ± 1.53 μm, followed by the consortium with 8.33 ± 2.08 μm, and the lowest of 6.67 ± 2.08 μm by the isolate Cyn2 (**Figure 2**).

### Tolerance of *Bacillus* spp. to abiotic stress

#### Temperature stress

**Figure 3:**
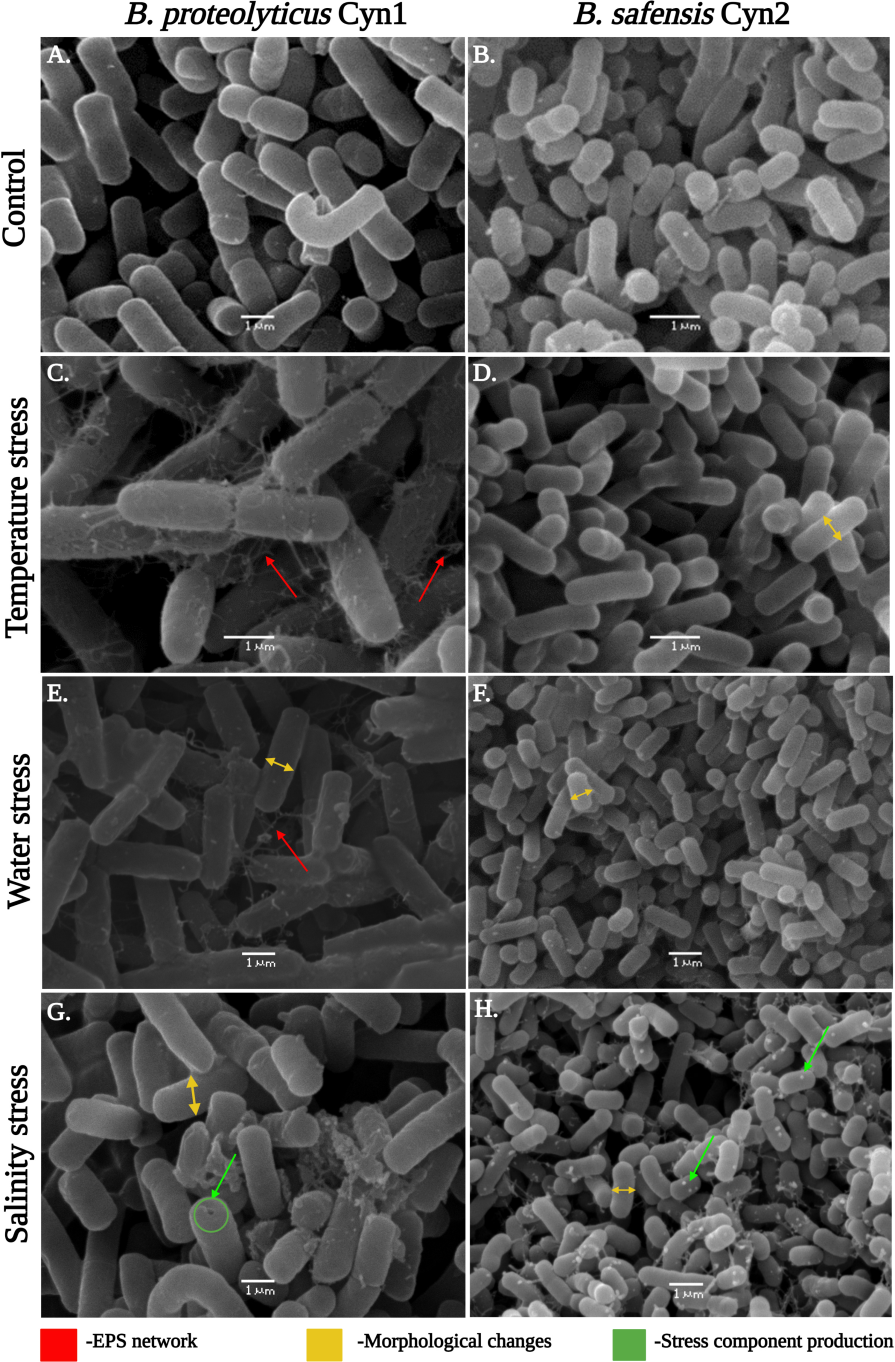
Scanning electron micrographs showing the cells of **(A, C, E, G)** *B. proteolyticus* Cyn1 and **(B, D, F, H)** *B. safensis* Cyn2 under temperature, drought, and saline conditions in comparison with the control.

**Figure 4:**
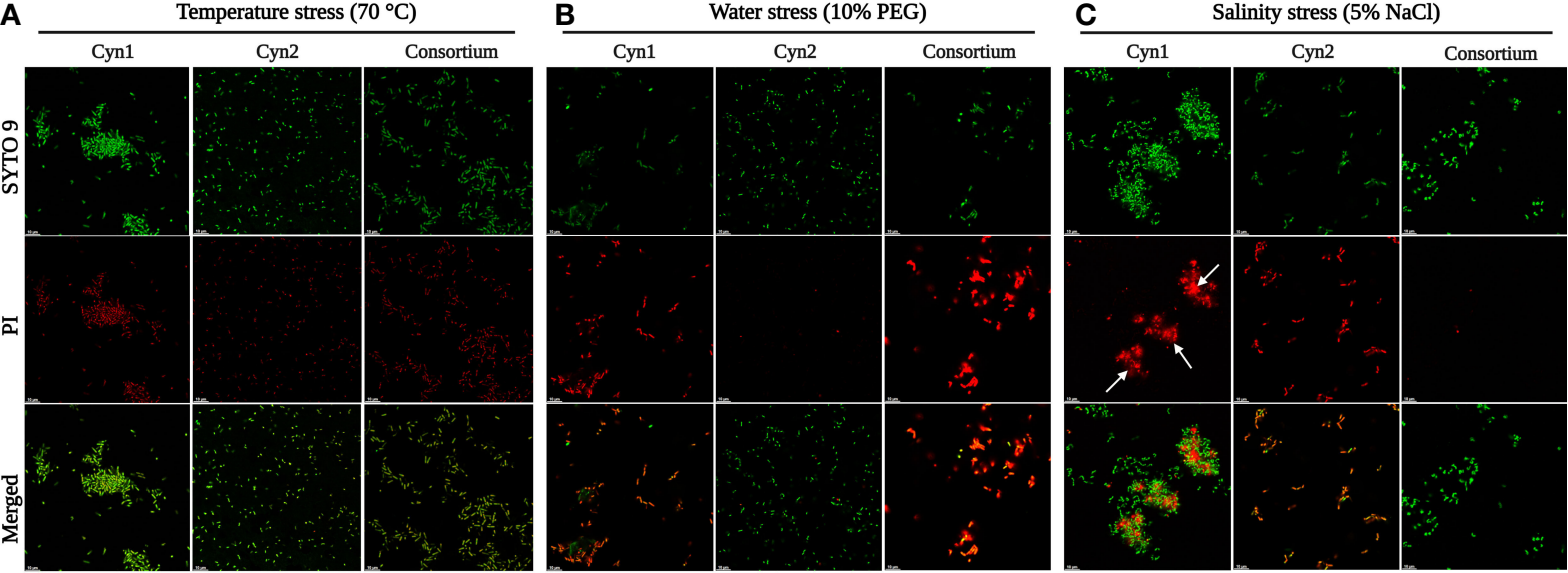
Cellular viability analyses of *B. proteolyticus* Cyn1 and *B. safensis* Cyn2 under different abiotic stresses *via* confocal microscopy, where **(A)** temperature stress, **(B)** water stress, and **(C)** salinity stress. The white arrows indicate the presence of eDNA.

As can be observed for the tolerance assay against abiotic stress temperature, both the isolated rhizospheric soil PGPB isolates Cyn1 and Cyn2 can tolerate temperature stress significantly. *B. proteolyticus* Cyn1 can tolerate a temperature treatment up to 80°C, while the bacterium *B. safensis* Cyn2 can tolerate temperature treatment up to 70°C (**Figure 3**). In the scanning electron micrographs, Cyn1 can be found to form surface polysaccharide networks at a higher temperature of 80°C. On confocal micrograph, both the isolates Cyn1 and Cyn2 under temperature stress were observed to be completely viable emitting significantly green fluorescence. On the other hand, in the case of the consortium, a similar kind of high cellular viability was observed for both Cyn1 and Cyn2 cells (**Figure 4A**).

#### Water stress

From the results of the abiotic “water” stress tolerance experiment, both the isolated PGPB *B. proteolyticus* Cyn1 and *B. safensis* Cyn2 grew up to an induced drought intensity of 10% PEG. Cyn1 was found to form a surface polysaccharide network to a smaller extent under water-stressed conditions as observed in the scanning electron micrograph (**Figure 3**). The polysaccharide network was significantly lower compared to the temperature stress. However, both the isolates showed shrunken cellular morphology with decreased diameters under introduced drought or water-stressed conditions. Interestingly, in the case of confocal microscopy analyses, a higher amount of viable cells were only found in the case of Cyn2 with high emission of green fluorescence, compared to the other two (Cyn1 and consortium), where a relatively higher number of cells showed red fluorescence indicating possible membrane structure alteration or damage under water stress (**Figure 4B**). The results also indicated inefficiency or absence of synergism of the consortium under water stress with more red fluorescence emission than green fluorescence (**Table 2**).

**Table 2:** Determination of plant growth indexes of Cyn1, Cyn2, and their consortium on *P. vulgaris* L. under salinity stress in comparison to control.

Growth indexes	Cyn1 H_2_O	Cyn2 H_2_O	Control H_2_O	Consortium H_2_O	Cyn1 NaCl	Cyn2 NaCl	Control NaCl	Consortium NaCl
**Total length**	17.0 ± 1.7	18.7 ± 2.5	10.3 ± 4.7	21.7 ± 3.1	16.0 ± 3.0	11.5 ± 4.4	12.3 ± 9.7	20.3 ± 2.1
**Plumule length**	6.2 ± 1.0	4.2 ± 1.4	3.3 ± 0.8	17.0 ± 2.6	5.2 ± 3.9	3.7 ± 0.8	5.5 ± 6.1	6.3 ± 1.2
**Radicle length**	8.0 ± 0.5	12.2 ± 4.2	5.0 ± 3.3	9.7 ± 3.0	8.8 ± 3.2	7.5 ± 4.4	6.0 ± 3.5	9.8 ± 1.0
**Root length**	8.0 ± 1.0	12.8 ± 4.5	6.7 ± 3.8	10.3 ± 1.2	10.5 ± 2.2	8.2 ± 4.2	6.8 ± 3.3	10.3 ± 1.5
**Number of roots**	10.3 ± 1.2	9.7 ± 4.2	12.0 ± 3.0	21.0 ± 1.0	11.7 ± 2.1	12.0 ± 2.0	10.0 ± 3.0	18.7 ± 4.0
**Shoot length**	1.1 ± 0.1	1.2 ± 0.3	1.0 ± 0.1	1.2 ± 0.3	1.6 ± 0.4	1.3 ± 0.5	1.4 ± 0.5	2.0 ± 0.0
**Number of leaves**	4.0 ± 0.0	2.7 ± 2.3	0.7 ± 1.2	4.0 ± 0.0	4.0 ± 0.0	4.0 ± 0.0	1.3 ± 2.3	4.0 ± 0.0
**Leaves width**	1.6 ± 0.2	1.3 ± 1.1	0.3 ± 0.6	1.8 ± 0.2	1.8 ± 0.3	1.8 ± 0.1	0.6 ± 1.0	1.8 ± 0.3
**Leaves length**	1.9 ± 0.1	1.0 ± 0.8	0.3 ± 0.4	1.4 ± 0.6	1.2 ± 0.1	1.3 ± 0.1	0.3 ± 0.5	1.8 ± 0.3
**Relative germination (%)**	100%	100%	100%	100%	100%	100%	100%	100%
**% Toxicity**	−	−	0%	−	−	−	0%	−
**Tolerance Index**	1.2	1.9	1.0	1.6	1.6	1.2	1.0	1.5
**Vigor Index**	880	10	1536	670	1236	1680	952	2060

“**−**” means not present.

#### Salinity stress

**Figure 5:**
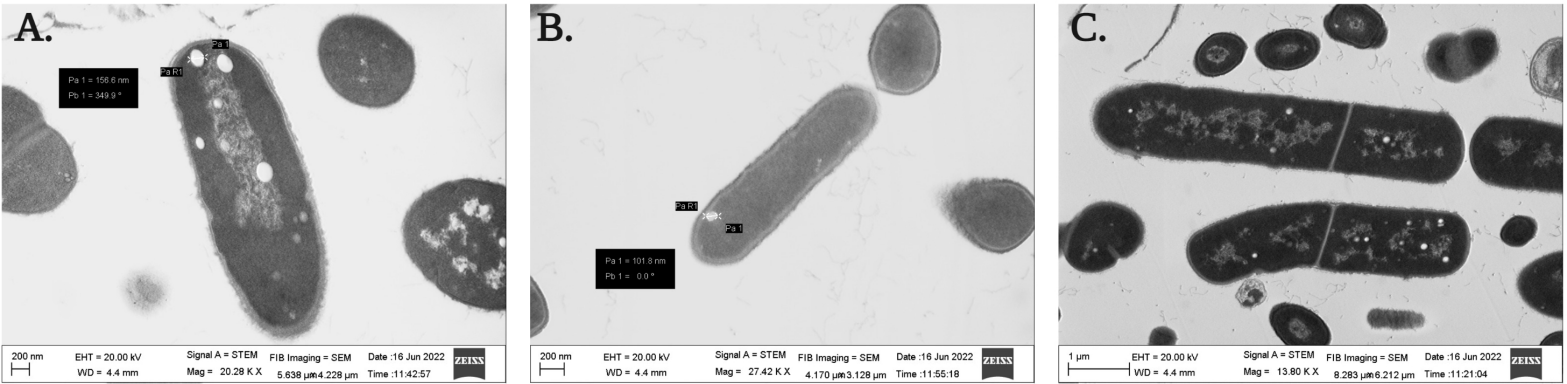
Transmission electron micrograph showing the formation of cytoplasmic inclusion bodies under salinity stress of **(A)** *B. proteolyticus* Cyn1, **(B)** *B. safensis* Cyn2, and **(C)** consortium.

Both the isolated PGPB from rhizospheric soil of Chilean common bean can tolerate salinity stress up to a level; *B. proteolyticus* Cyn1 can tolerate a salinity stress of 5%, while the bacterium *B. safensis* Cyn2 can tolerate a salt concentration of up to 8%. In the scanning electron micrographs (**Figure 3**), Cyn1 was visibly swelled at high saline condition of 5% compared to the control condition and so did Cyn2. The production of some stress component may also be detected from the SEM images to mitigate the osmotic stress under high salinity. From the confocal micrograph of cell viability analyses under salinity stress, the PGPB isolates Cyn1 and Cyn2 emitted green and red fluorescence, respectively. In case of *B. proteolyticus* Cyn1, a bright green fluorescing cell was found on a red fluorescing bed, possible due to the presence of eDNA inside biofilms. The number of green fluorescence emitting cells, i.e., viable cells, were more in the case of Cyn1 than in the other two conditions (**Figure 4C**). However, the consortium demonstrated the best synergism in terms of the most brightly fluorescing green bacterial cells with no emission of red fluorescence, indicating the best cellular viability. Among all the three studied abiotic stresses, as the bacteria responded best under salinity stress, salinity stress has been chosen for further seed germination and plant growth study. From the transmission electron micrograph (**Figure 5**), the formation of small, cytoplasmic inclusion bodies within the cell have been observed for both *B. proteolyticus* Cyn1 and *B. safensis* Cyn2 under 5% and 8% salinity stress induction, respectively. While the size of the circular inclusion bodies for Cyn1 was 156.63 ± 5.55 nm, for Cyn 2, it was a little less (105.01 ± 9.18 nm). The inclusion bodies can be a possible tool of the bacteria to fight osmotic stress under salinity.

### Seed bacterization and germination study

**Figure 6:**
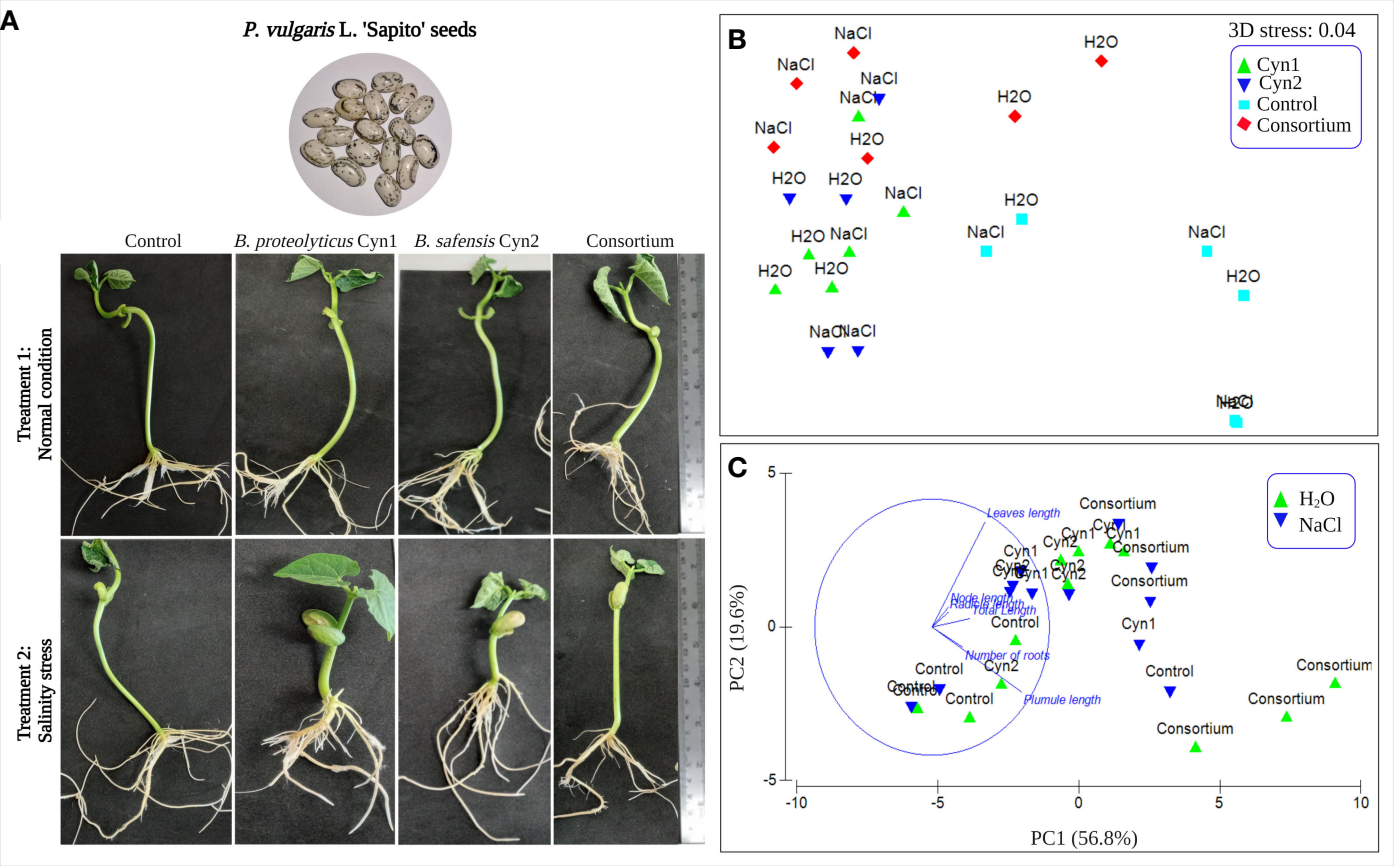
**(A)** Effect of seed bacterization on the growth of “Sapito” variety common beans, where treatment 1 is the normal condition and treatment 2 under introduced salinity stress (5%), respectively for control without any inoculation, and inoculation Cyn1, Cyn2, and consortium. **(B)** NMDS plot with a 3D stress of 0.04. **(C)** Principal component analysis results loading plot PC1 (56.8%) and PC2 (19.6%) variances.

The influence of the two strains alone and the consortium of the two isolates on the growth promotion of *P*. *vulgaris* L. “Sapito” seeds under normal and salinity stress conditions was evaluated *via* seed bacterization and seedling germination. While compared to the control condition, seeds inoculated with *B. proteolyticus* Cyn1 under salinity stress (5% NaCl) showed less growth, the inoculation of *B. safensis* Cyn2 to the seeds under similar salinity stress showed increased growth than the control. In the case of the consortium, the seeds treated with 5% salt concentration showed overall better growth than seeds with single bacterial treatment (**Figure 6A**). The germination index was 100% for both the experiments under normal condition and salinity stress. The number of roots were higher in the case of the consortium for both experimental setups. The height of the shoot of the germinated seedlings were observed to be highest for the experimental setup 1 containing Cyn2 followed by the setup with NaCl containing consortium.

Furthermore, the *in vitro* root–bacteria association study performed 5 days post-bacterization showed all the parts of the root system colonized by the PGPB strains and their consortium. Visually, under treatment 1 in normal condition, a higher cellular density on root surfaces was observed for *B. proteolyticus* Cyn1 followed by the consortium. Compared to treatment 1, in treatment 2 subjected to 5% salinity stress, a higher cellular density or root colonization was observed for the consortium followed by *B. proteolyticus* Cyn1; Cyn2 in both experiments showed visibly less root colonization.

The PERMANOVA test showed a significant p (perm)-value of 9e−5. Non-metric multidimensional scaling (NMDS) plot of the experiments demonstrated good quality of ordination, having a low 3D stress value of 0.04, whereas the bacterial isolates *B. proteolyticus* Cyn1 and *B. safensis* Cyn2 did not demonstrate any significant differences in the germination parameters. However, the consortium and control showed to be significantly different (**Figure 6B**). As it may be observed from the PCA analyses (PC1 = 56.8% and PC2 = 19.6% as shown in **Figure 6C**), the distance between the leaf length and plumule length is highest from the control. Thus, under salt stress, the plant produces less plumule and more leaves, and without salt stress, they are longer in size.

### Pot experiments

**Figure 7:**
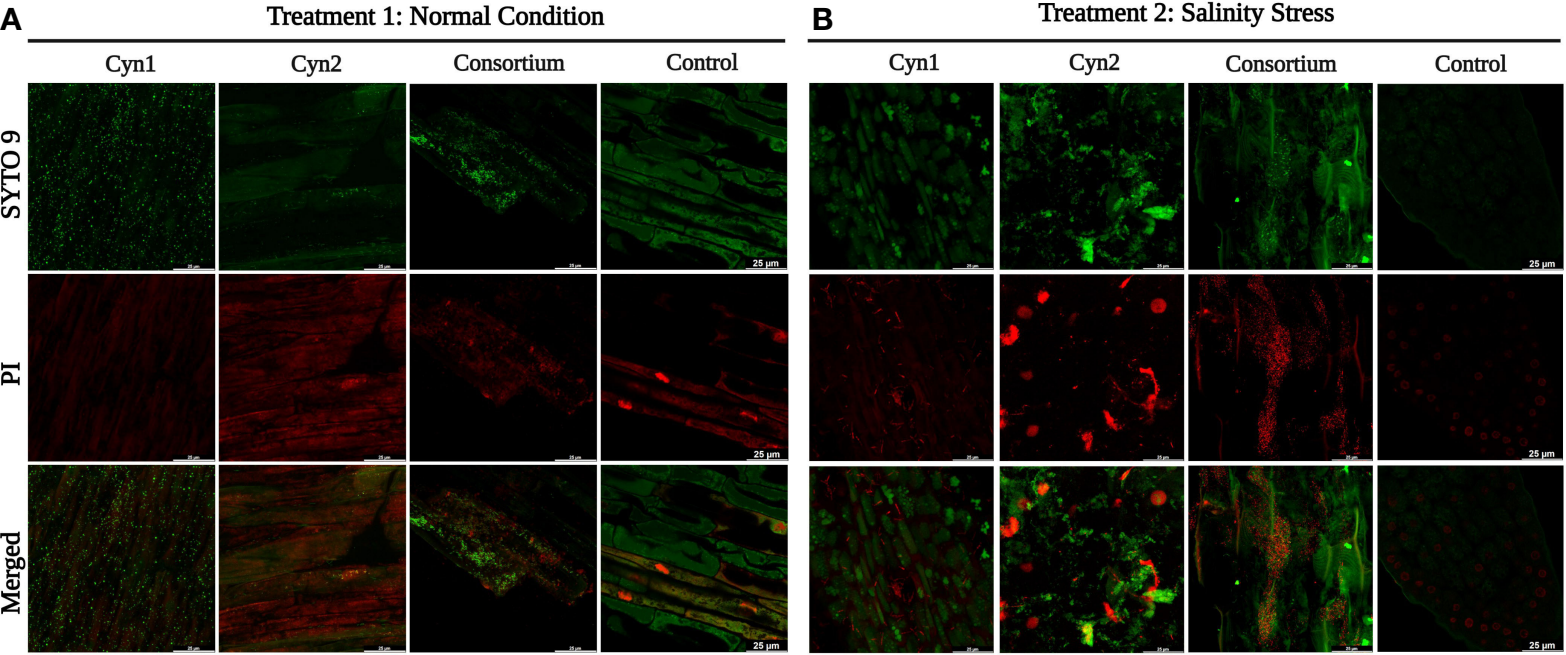
Confocal microscopy images showing root–bacteria association. Staining was done with SYTO 9/propidium iodide (LIVE/DEAD BacLight Bacterial Viability kit), and images were acquired at 60 x magnification. **(A)** Treatment 1: normal condition. **(B)** Treatment 2: salinity stress.

**Figure 8:**
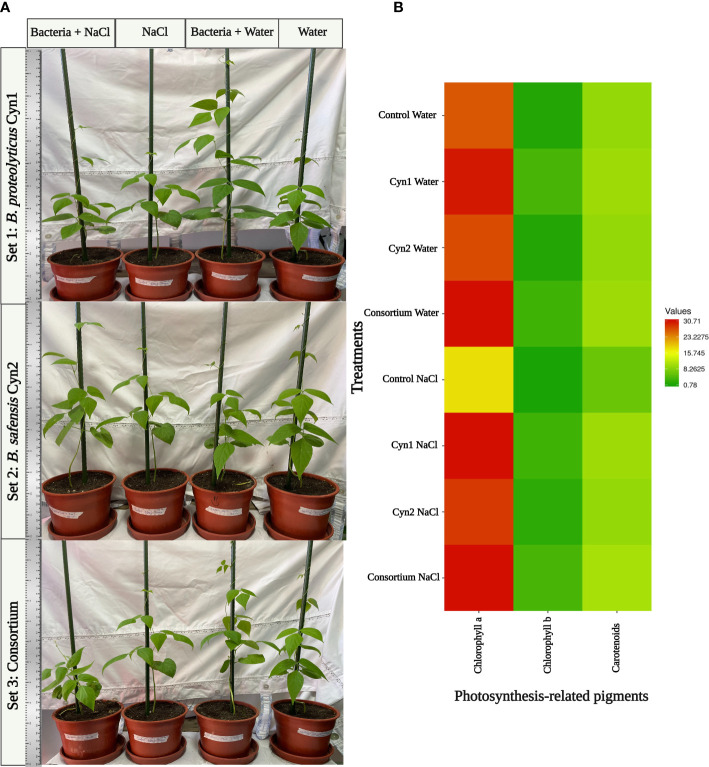
**(A)** Evaluation of *B. proteolyticus* Cyn1, *B. safensis* Cyn2, and their consortium as candidates for the growth of Chilean local “Sapito” variety of common beans under normal conditions and induced salinity stress in pot experiments. **(B)** Photosynthesis-related parameters chlorophyll a, chlorophyll b, and total carotenoids content of the treated plants after 28 days of growth.

The influence of the two strains alone and the consortium in growth promotion of *P*. *vulgaris* L. under normal and 5% salinity stress conditions was evaluated by pot experiments. In general, salinity adversely affects the plant productivity. However, plants bacterized with *B. proteolyticus* Cyn1 and the consortium of both Cyn1 and Cyn2 demonstrated visibly higher growth under normal condition compared to the control plant without any root bacterial colonization. In the case of the consortium, bacterized plant obtained a little more growth under normal conditions compared to the control (**Figure 7A**). In comparison, plants treated with *B. safensis* Cyn2 demonstrated better growth under 5% salinity and salinity-induced osmotic stress compared to the control plants under salinity stress and no root bacterial colonization (**Figure 7B**). From the results of the photosynthetic pigment measurements after a month of pot experiment (**Figure 8A**), the presence of chlorophyll a pigment was highest when the plants were treated with the consortium and *B. proteolyticus* Cyn1 in both normal condition and salinity stress (**Figure 8B**). Additionally, a similar trend is observed for the other two photosynthetic pigments: chlorophyll b and carotenoids, too. It has been interestingly observed that the results of the photosynthetic pigment measurement showed how plants bacterized with both the isolated PGPB, i.e., the consortium alone synergized in obtaining an improved photosynthetic pigment production when subjected to salt stress (**Supplementary Table S1**).

## Discussion

Cereals and legumes are grain-producing crops that provide more than half of the total human protein and energy required. Many studies have been performed on wheat and rice, whereas there is a lack of knowledge about salt tolerance in cereals (Shahbaz and Ashraf, 2013). In general, common bean is sensitive to salt. It is actually reported to be a glycophyte, i.e., sensitive to salt, and even at low soil salinity<2 dS m^−1^, a significant reduction in crop productivity happens (Al Hassan et al., 2016). However, at the time of cultivation of this crop, protein concentration was found to be increased with increasing salinity (Bayuelo-Jiménez et al., 2022). At the same time, due to the high demand of vegetable proteins, the area cultivated with this crop is expected to increase. As saline soils are also likely to surge, it is important to look for strategies to cultivate common beans without affecting yields. In this study, salt tolerance of one Chilean common bean ecotype “Sapito” (*P*. *vulgaris* L.) has been studied, which makes this study innovative, as this kind of study on Chilean common bean has not been reported before. For this experiment, native bacteria from rhizospheric soil of *P*. *vulgaris* L. have been isolated and checked for their PGPB activity, abiotic stress tolerance (temperature, drought, and salinity), and plant–microbe interaction under both normal condition and induced salinity stress.

Two free, soil living bacteria, *B*. *proteolyticus* Cyn1 and *B*. *safensis* Cyn2, were isolated from rhizospheric soil samples of *P. vulgaris* L. (**Figure 1**). *B. proteolyticus* Cyn1 can produce NH_3_ and HCN, and also can secrete siderophore, whereas *B*. *safensis* Cyn2 can produce NH_3_ and siderophore but cannot produce HCN like Cyn1. The secretion of NH_3_ increased the alkalinity of the soil by increasing the pH, which inhibited the growth of many plant pathogenic fungi and even fungal spore formation. In this way, NH_3_ production improves the crop yield *via* maintaining the crop health (Swamy et al., 2016; Richard et al., 2018; Gupta and Pandey, 2019). It actually supplies the macronutrient nitrogen to the host plant and thereby promotes root and shoot elongation and improved biomass, making it as one of the most critical PGPB character (Bhattacharyya et al., 2020). Siderophore production is another crucial PGPB trait that indirectly influences plant growth. To protect plant health, low molecular weight siderophores chelate available Fe^3+^ and other metals present in the rhizospheric region to make them unavailable to the phytopathogens, hence maintaining the plant health (Joseph et al., 2007; Ahmad et al., 2008). By this mechanism, siderophore also contributes to disease suppression, including stimulating the local and systematic host resistance to biosynthesize some antimicrobial compounds, which may otherwise act as biotic stress factor to the plant (Joseph et al., 2007). In this study, both isolated PGPB *B*. *proteolyticus* Cyn1 and *B*. *safensis* Cyn2 are positive ACC deaminase producer. ACC deaminase is a repressor for stress ethylene production by breaking down the immediate precursor of ethylene under different biotic and abiotic conditions, as it acts as a growth inhibitor (Singh et al., 2015). Bacteria with positive ACC deaminase activity can increase the tolerance to abiotic stress in various plants, i.e., bacteria can promote plant growth under different environmental stress conditions (Singh et al., 2015). Being positive responders, studied PGPB isolates Cyn1 and Cyn2 can also be used as plant growth promoters for future agriculture. Additionally, both the isolated PGPB Cyn1 and Cyn2 were showing positive result for catalase enzyme production. Catalase enzyme plays a key role in the acclimation process of plants to different abiotic stresses, as they provoke the formation of reactive oxygen species (ROS). Being an antioxidant enzyme, catalase acts in stress metabolism and reduces the toxicity of ROS (Babu and Devaraj, 2008; Choudhury et al., 2017). Additionally, in this study, both the PGPB isolates have been observed to be excellent solubilizer of inorganic phosphates. Phosphorus is another essential macronutrient for the plant, but most of the time, it remains unavailable to the plants. For this, chemical fertilizer is used excessively as a source of inorganic phosphorus in regular agricultural practices, leading to lethal consequences for beneficial microbes and soil pollution (Naik et al., 2008). Microorganisms can solubilize the insoluble organic phosphate *via* breaking the ester bond by which phosphate gets bound to the soil organic matter, making them available to plants to maintain their health (Richardson, 2001). Positive phosphate solubilization may lead the bacteria to be used as biofertilizers in the near future. Furthermore, *B*. *proteolyticus* Cyn1 being the HCN producer has increased availability of phosphates. HCN not only acts as a biocontrol agent to the plant pathogens in rhizosphere but also is reported to be involved in geochemical processes in the substrate, specifically chelation of metals, and hence indirectly increasing the availability of phosphates to the plant resulting in improved plant growth (Rijavec and Lapanje, 2016). Finally, it is important to mention that both the PGPB isolates and their consortium are biofilm formers, where the cells remain viable, maintaining their respective bioactivities. Biofilm formation is an indirect PGP trait of the soil bacteria, as they form a microenvironment maintaining the ecological balance and plant growth in both the natural soil and soil with distinct abiotic stresses like desiccation, elevated salt/metal content, increased temperature, and more (Kasim et al., 2016). Therefore, the soil bacilli in our study have the efficiency to demonstrate several PGP traits that can potentially help in plant growth in normal conditions and under stress.

Drought, salinity, and extreme temperatures are the principal abiotic stress factors affecting crop growth and productivity globally. Among all, salinity stress is considered one of the main challenges for contemporary agriculture because it affects the germination rate and reduces plant growth and crop productivity drastically (Negacz et al., 2022). It has been estimated that, worldwide, more than 1 billion hectares of land are affected by salinity (Qadir et al., 2014; Negacz et al., 2022). Consequently, global warming and climate change also lead to the expansion of semi-arid and saline lands, mainly in central and northern Chile (Martínez-Villavicencio et al., 2011). Bacteria inhabiting the root-associated region of the plants, i.e., rhizospheric bacteria, may have the ability to tolerate salinity and water stress. They might be used to prevent crop loss that happens due to abiotic stress. In this context in the present study, isolated bacteria Cyn1 and Cyn2 were used to investigate plant–microbe interaction. They have been found to have good tolerance against different abiotic stresses, *viz*., temperature (80°C and 70°C, respectively, by *B*. *proteolyticus* Cyn1 and *B*. *safensis* Cyn2), water (both till 10% PEG), and salinity (5% and 8% NaCl, respectively, by *B*. *proteolyticus* Cyn1 and *B*. *safensis* Cyn2) along with promising PGP activities as discussed before. From the scanning electron micrographs, it has been found that in comparison to Cyn2, Cyn1 produced strands of polysaccharides, making strong EPS network under temperature stress (**Figure 3**). Actually, a dense network of extracellular appendages of polysaccharides, a feature of biofilm formation, has been earlier reported for thermotolerant and thermophilic bacterial species, *viz.*, *Thermincola*, *Geobacter*, etc. (Parameswaran et al., 2013). Additionally, in drought stress, both the PGPB isolates have been observed to have significant morphological changes in terms of reduction in cell width with shrunken nature and presence of some EPS network in isolate Cyn1. This can be due to the maintenance of internal turgor pressure of the bacteria. In the case of salinity stress, both Cyn1 and Cyn2 have shown swollen cell morphology and formation of stress component within the cell (**Figures 3G, H**) as previously found by Bron et al. (2004). Furthermore, the transmission electron microscopic study confirmed the formation of small cytoplasmic inclusion bodies under salinity stress (156.63 ± 5.55 nm for Cyn1 and 105.01 ± 9.18 nm for Cyn2) (**Figure 4**). This kind of inclusion bodies actually contains Na^+^ ions accumulated due to salinity stress. The formation of polyhydroxyalkanoates (PHA) granules has also been observed before in these inclusion bodies to combat the stress condition. A similar kind of vacuole-like structure formation has been observed in *Halomonas pacifica* ASL10 and *Halomonas salifodiane* ASL11 isolated from Mariout salt lakes containing Na^+^ ions and PHA (El-Malek et al., 2020). Furthermore, cellular viability studies *via* confocal microscopy have been performed for these PGPB isolates under stress conditions. From the confocal micrographs, it has been observed that the bacterium *B. proteolyticus* Cyn1 has better viability to temperature and salinity stresses, having a significant number of live bacteria showing green fluorescence (**Figures 4A, C**). In the case of salinity stress, eDNA in the biofilm network can also be visualized (**Figure 3**). Morcillo and Manzanera (2021) already reported bacterial EPS to alleviate plant abiotic stress tolerance, including salinity, drought, temperature, and heavy metal toxicity. In the case of drought, EPS maintains a hydrated microenvironment surrounding the bacteria, reducing the water loss, which endorse bacterial survival in the extreme environment. For temperature stress, the surrounding network formed by the bacterial EPS around the plant roots improves water retention, improving the effects of heat shock in the plants (Morcillo and Manzanera, 2021). In the case of salt tolerant bacteria, they adapt a few mechanisms related to the cell wall structure, ion/proton pumps, etc. that make them survive under salt stress. Bacteria may produce EPS limiting the salt import within the cell and optimizing the Na^+^/H^+^ pump within the cell wall (Shilev, 2020). In an earlier study, it has already been reported that endophytic *B*. *safensis* ZY16 can be used for improved phytoremediation of 0.8% saline soils (Wu et al., 2019). Under salinity stress, bacteria were found to synthesis stress component.

In the case of seed bacterization and germination experiment, *B*. *safensis* Cyn2 showed good response in normal condition, whereas under salt stress, *B*. *proteolyticus* Cyn1 expressed improved response (**Figure 6**). Furthermore, the cell count was observed to be high in plant root when treated with Cyn1 and consortium of both. Germinated seedlings were used further to change to pot experiment. Pot experiment is considered as complement of field study, as it allows to perform the experiments on growth of plant under controlled and desired condition without a heterogeneous effect of different environmental factors in different seasons (Kawaletz et al., 2014). Considering the two experimental set ups in normal condition and salinity stress, the effect of *B*. *proteolyticus* Cyn1 and the consortium of both Cyn1 and Cyn2 was found to be best improving the growth of the common bean. Increased root numbers for bacterized plants growing under salinity stress were probably because under salinity stress, the bacteria are accumulating in the root to combat the stress followed by improved growth. According to one previous study, it has been found that due to salt stress, root colonization of *B*. *amyloliquefaciens* NBRISN13 in rice plants was increased, followed by increased production of ACC deaminase to maintain the chlorophyll content of the plant (Numan et al., 2018), which is due to the bacterial activity against abiotic stress to help the plant grow healthy. In the present study, similarly, chlorophyll and carotenoid pigment contents were found to be increased significantly under salinity stress for the treatment with *B*. *proteolyticus* Cyn1 and the consortium (**Figure 7**). Significantly, both the bacteria were found to be ACC deaminase positive in our study. Alongside, both the PGPB isolates are catalase positive, too, which denotes amelioration of the salinity-stress-related toxicity as previously found by Babu and Devaraj (2008) in French beans. Thus, from this result, it can be clearly understood that both the PGPB isolates and their consortium successfully demonstrated plant-growth-promoting activity and helped in the elimination of the toxic effect of salt stress that resulted in increased root length, improved vigor, and more photosynthetic pigment production. A recent study by Wang et al. (2018) on *Bacillus* spp. also revealed high ACC deaminase activity, siderophore production, and phosphate solubilization, leading to amelioration of salt stress similar to our study. Thus, our present study indicates the result of improved plant–bacteria interaction in the Chilean “Sapito” ecotype of *P*. *vulgaris* L., which promoted successful seed bacterization followed by improved germination rate and seedling development to healthy plant growth alleviating the harmful effect of salinity. As both the isolated PGPB *B*. *proteolyticus* Cyn1 and *B*. *safensis* Cyn2 have shown healthy and improved plant growth related to their different plant-growth-promoting traits and abiotic stress tolerance and helped to grow healthy common bean plant by decreasing the level of toxicity under salinity stress; hence, these isolated bacteria can be used in circular bioeconomy in the near future in the agricultural industry.

## Conclusion

Plant–microbe interaction plays a basic yet crucial function in improving crop yield by establishing new strategies without using chemical fertilizers. The symbiotic interaction of soil bacteria colonizing the rhizosphere helps in improved nutrient and minerals uptake, inhibition of plant pathogens, and creation of a microenvironment around the root under different abiotic stresses that in turn helps in the healthy growth plants. In this study, both the isolated PGPB *B*. *proteolyticus* Cyn1 and *B*. *safensis* Cyn2 were positive for ACC deaminase and catalase enzyme production, phosphate solubilization, siderophore production, and biofilm network formation. Additionally, Cyn1 was an HCN producer, too. They both have demonstrated significant temperature, drought, and salinity stress tolerance. Present experimental data and evidence demonstrated successful seed germination after seed bacterization of “Sapito” under 5% salt stress. Root colonization of bacteria may have reduced the toxic effect of salt stress. In addition, both bacteria have responded with good PGP traits that helped in the growth of healthy plants after the bacterial treatment. Healthy and improved growth of *P*. *vulgaris* L. have been observed in both normal and salinity stress conditions, indicating successful plant–bacteria interactions. As PGPB serves multiple purposes, *viz*., reduction in chemical fertilizers usage, improved soil health, enhanced crop yield, and biofortification in commercial agricultural practices, these bacterial isolates or their consortium can be used in circular economy to fight plant abiotic stresses in the near future along with improved agricultural production of Chilean native common beans.

## Data availability statement

The original contributions presented in the study are included in the article/**Supplementary Material**. Further inquiries can be directed to the corresponding authors.

## Author contributions

CM, FV, AG, and AB conducted the experiments. CM, AB, and BC analyzed the experimental data. AE performed the statistical analyses. AB collected the samples and conceptualized the work. CM, SS, and AB wrote the first draft of the manuscript. AB, SS, RC, BC, AA, and KQ reviewed, edited, and finalized the manuscript. BC obtained the funding. All the authors contributed to the article and approved the submitted version.

## Supplementary material

Supplementary Figure 1 LB plate showing mucoid colony appearance of **(A)** *B. proteolyticus* Cyn1 and **(B)** *B. safensis* Cyn2; (C) Comparison of different plant growth promoting characters of PGPB isolates Cyn1 and Cyn2.
