# Supplementary material for: Plant-growth-promoting bacteria from rhizosphere of Chilean common bean ecotype (Phaseolus vulgaris L.) supporting seed germination and growth against salinity stress
Source: Front Plant Sci. 2022 Dec 22;13:1052263. doi: 10.3389/fpls.2022.1052263 (PMC9814130; doi:10.3389/fpls.2022.1052263)
Supplement: Supplementary file 1 [file Table_1.docx]

**Table S1.** Photosynthetic pigment production by Chilean ecotype (Sapito) of *P. vulgaris* L. under salinity stress in comparison to control condition showing the effect of bacterization with *B*. *proteolyticus* Cyn1, *B*. *safensis* Cyn2 and their consortium (n=3)

| **Photosynthetic pigments**  **(Absorbance)** | **Experiment 1: Normal Condition** | | | | **Experiment 2: Salinity Stress** | | | |
| --- | --- | --- | --- | --- | --- | --- | --- | --- |
|  | **Control H_2_O** | **Cyn1 H_2_O** | **Cyn2 H_2_O** | **Consortium H_2_O** | **Control NaCl** | **Cyn1 NaCl** | **Cyn2 NaCl** | **Consortium NaCl** |
| **Chlorophyll a (OD_662 nm_)** | 2.1720 ± 0.0075 | 2.4926 ± 0.0264 | 2.1163 ± 0.0335 | 1.4227 ± 0.0176 | 2.4580 ± 0.0096 | 2.5170 ± 0.0492 | 2.5073 ± 0..0015 | 2.2877 ± 0.0146 |
| **Chlorophyll b (OD_646 nm_)** | 0.9613 ± 0.0057 | 1.1547 ± 0.0167 | 0.9293 ± 0.0237 | 0.6187 ± 0.0075 | 1.1717 ± 0.0071 | 1.1867 ± 0.0116 | 1.1617 ± 0.0045 | 1.0397 ± 0.0091 |
| **Carotenoids (OD_470 nm_)** | 2,.5723 ± 0.0142 | 2.9487 ± 0.0345 | 2.5870 ± 0.0412 | 1.6727 ± 0.0275 | 3.0160 ± 0.0140 | 3.0533 ± 0.0673 | 3.1467 ± 0.0191 | 2.7393 ± 0.0248 |

´n´ means the number of experimental data collected in replicates
