# Supplementary figures and images for: Plant-growth-promoting bacteria from rhizosphere of Chilean common bean ecotype (Phaseolus vulgaris L.) supporting seed germination and growth against salinity stress
Source: Front Plant Sci. 2022 Dec 22;13:1052263. doi: 10.3389/fpls.2022.1052263 (PMC9814130; doi:10.3389/fpls.2022.1052263)

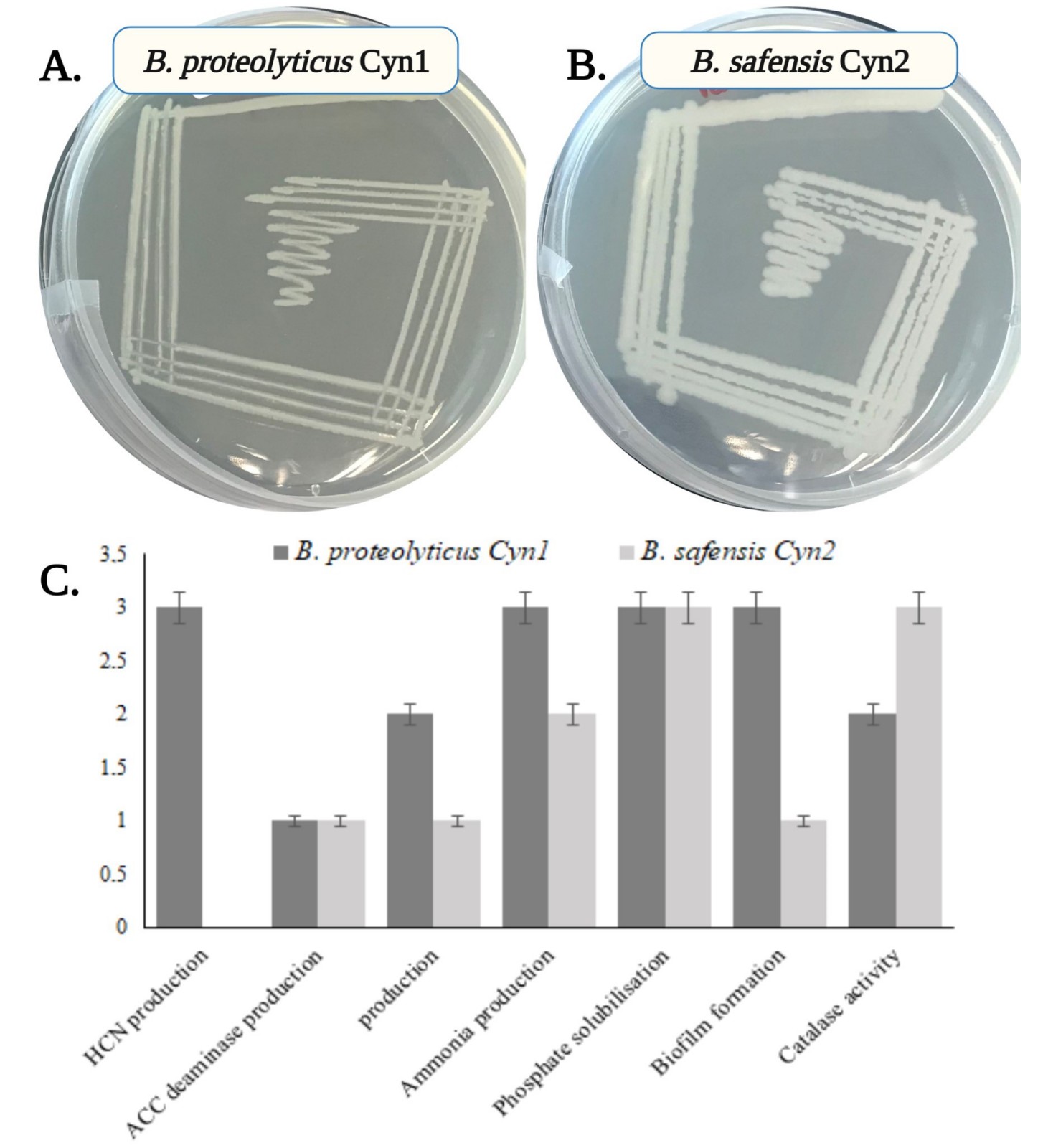

Supplement: Supplementary Figure 1 — LB plate showing mucoid colony appearance of (A) B. proteolyticus Cyn1 and (B) B. safensis Cyn2; (C) Comparison of different plant growth promoting characters of PGPB isolates Cyn1 and Cyn2. [file Image_1.jpeg]
